# Research questions in pre-hospital trauma care

**DOI:** 10.1371/journal.pmed.1002345

**Published:** 2017-07-18

**Authors:** David J. Lockey

**Affiliations:** 1 Blizard Institute, Queen Mary University, London, United Kingdom; 2 London’s Air Ambulance, Royal London Hospital, London, United Kingdom

## Abstract

Noting that a variety of pre-hospital interventions can now be applied to treat traumatic injury, David J Lockey calls for research to determine which of these actually improve survival and reduce morbidity.

## The evidence base for pre-hospital trauma care

Twenty years ago, an editorial examining the evidence for, and worldwide expenditure on, pre-hospital emergency care noted that—despite considerable expenditure—the evidence base for the field was less than that of urticaria or constipation [1]. Much has been published since, but many key questions about the provision of early trauma care still have to be addressed [2].

Most advanced EMS (Emergency Medical Service) systems are designed to identify seriously injured trauma patients and transport them rapidly to a suitable hospital. Many also dispatch advanced clinical teams with extended skills to the scene. These teams provide interventions to either treat time-critical pathology or to avert deterioration or death before in-hospital interventions are available. In low- and middle-income countries, EMS infrastructure rarely allows advanced on-scene care, and basic infrastructure improvements (e.g., effective ambulance services) are a higher priority.

Advanced trauma systems are inclusive—they quality assure the whole trauma care pathway from point of injury to rehabilitation. This approach encourages parallel development of pre- and in-hospital services and increases attention on the quality of care outside the major trauma centre, usually the priority site for trauma care initiatives. “Early” trauma care interventions are often described as commencing in the receiving emergency department, but as hospital survival improves an increasing proportion of trauma deaths occur in the pre-hospital phase of care. Despite this, significant pre-hospital trauma research with potential for improving outcomes remains limited. Most pre-hospital trauma research studies are based on trauma registry data, sometimes using prospective or before-and-after study designs, and randomised trials are rare. Studies have usually focussed on which interventions deliver benefit and how they can be delivered.

## What can be delivered currently?

In Europe, where pre-hospital care is frequently physician-delivered, advanced pre-hospital critical care interventions are often performed at the scene of the injury, and new interventions are reported regularly. Advanced interventions are less commonly reported in the United States, where pre-hospital care is delivered by nonphysicians. Pre-hospital anaesthesia, mechanical ventilation, chest decompression, resuscitative thoracotomy, and advanced monitoring have been performed for many years. More recently reported interventions include pre-hospital transfusion [3], extracorporeal membrane oxygenation [4], CT scanning with stroke thrombolysis [5], resuscitative endovascular balloon occlusion of the aorta [6], and point-of-care testing (e.g., ultrasound imaging, blood gas analysis, plasma lactate, and tissue oxygen saturation). This wide range of interventions indicates that, with sufficient resources and training, most in-hospital interventions can be delivered before hospital arrival.

The delivery of advanced interventions on scene is promoted on the basis that, if left untreated until hospital arrival, some patients will die or suffer morbidity from uncorrected time-critical pathology. The in-hospital intensive care principle of “critical care without walls” [7]—that patients should be treated on the basis of their compromise rather than their geographical location—can similarly be applied to pre-hospital trauma care. Airway compromise, haemorrhage and tension pneumothorax have been the focus of most time-critical interventions to date. Now that a wide range of interventions that can be applied in the pre-hospital phase of care has been established, the more difficult question of which interventions should be performed is the research challenge for the immediate future.

## Key questions

True innovation is rare in pre-hospital trauma care. Most promising interventions are developed in-hospital and, where practical, translated into the pre-hospital phase of care. Major breakthroughs in pre-hospital trauma care are likely to follow the same breakthroughs in in-hospital practice—effective blood substitutes and haemostatic and neuroprotective techniques are, for example, aspirations in both areas. There are exceptions to this, and some technological advances may only be applicable in the pre-hospital phase of care. Examples include vehicle automatic crash notification technology or the use of unmanned aerial vehicles for surveillance or for the rapid delivery of equipment or interventions to the scene of injury.

Although surgical interventions receive most attention, they are the most difficult to introduce into routine pre-hospital practice, and less invasive interventions may also have the potential to improve outcomes. Technological advances make many modes of monitoring and investigation readily available to pre-hospital care providers. Diagnostic ultrasound and tissue oxygen saturation [8] are 2 examples. Pharmacological pre-hospital interventions are straightforward to administer; tranexamic acid [9] is an example of a drug reported to provide benefit when administered as early as possible after insult. Anticoagulant reversal after head injury [10] is a more recent pharmacological pre-hospital intervention. Other agents have the potential to modify the response to hypoperfusion and prevent organ damage when administered early after injury [11].

Studies in the pre-hospital phase of care may improve our understanding of the pathophysiology of hyperacute injury and yield new knowledge of which interventions might influence outcomes, and by what mechanisms. Some early mortality may only be reduced by accident prevention or mitigation techniques, but to reduce mortality on scene in those who are seriously injured requires a deeper understanding of early injury pathophysiology, the timelines of deterioration, markers of severity, and the requirement for early intervention. Current physiological markers, e.g., heart rate, blood pressure, and plasma lactate [12] are insensitive and often give little indication of which patients will deteriorate quickly. Inflammatory markers have been described as possible indicators of injury severity and prognosis but are not yet available for practical use in resuscitation.

Once efficacy has been established, the question of how interventions should be delivered is more about pre-hospital service infrastructure than the interventions themselves. Although the principles of the US model of trauma care have been influential in many countries, there is surprising variation and disagreement about how care should be delivered. Provider levels, transport methods, and triage and hospital bypass guidelines are all the subject of controversy and still require investigation to maximise benefit in resource-limited systems. In cardiac arrest research early identification, dispatcher advice and instructions to bystanders have been identified as key factors in progress [13]. Not all trauma patients have the same treatment urgency as victims of medical cardiac arrest, but many of the concepts and priorities are the same.

## Barriers to conducting pre-hospital research

The barriers to effective pre-hospital trauma research are substantial. Trials involving acutely injured patients who cannot consent to treatment or trial recruitment are possible but arduous. Even well-designed randomised trials usually compromise to achieve recruitment, for example in using randomisation by provider, vehicle, or region rather than of patients [14]. The lack of standardisation in case mix, providers, timelines, and endpoints can make generalisability of results difficult. Patients with very severe injuries tend to have many interventions during their hospital stay which may vary both in, and between, trauma centres. Mortality may therefore be an insensitive outcome measure of an isolated pre-hospital intervention. Pre-hospital trauma registry studies are increasingly performed with larger patient numbers [15], but patient heterogeneity, inconsistency in the care provided, and incomplete data make interpretation of this type of study difficult—particularly with mortality as the primary outcome. Even well-funded studies can encounter major limitations when service providers do not recruit and deliver interventions consistently [16].

## Conclusion

Pre-hospital trauma care is a relatively undeveloped research area. Many advanced interventions are now possible, and future studies need to establish which improve survival and reduce morbidity. Advanced surgical interventions are likely to target a small number of severely injured patients in high-income countries but have shown promise. In-hospital trauma mortality is declining, and research to target accident prevention and reduce the mortality of patients who die before reaching hospital is key to reducing overall trauma mortality.
